# Oculocardiac Reflex in a Patient With Maxillofacial Trauma: A Case Study and Literature Review

**DOI:** 10.7759/cureus.59528

**Published:** 2024-05-02

**Authors:** Ayush Kharia, Srerama Janardhana Rao, Vertika Dubey, Sumit Bhatt, Drishti Bhatt, Fawaz Baig

**Affiliations:** 1 Department of Oral and Maxillofacial Surgery, People's College of Dental Sciences and Research Center, Bhopal, IND; 2 Department of Dental Surgery, Government Medical College, Srikakulam, IND; 3 Department of Oral and Maxillofacial Surgery, Private Dental Clinic, Bhopal, IND; 4 Department of Oral and Maxillofacial Surgery, Rajasthan Dental College and Hospital, Jaipur, IND; 5 Department of Oral and Maxillofacial Surgery, Divya Jyoti College of Dental Sciences and Research Centre, Modinagar, IND; 6 Department of Oral and Maxillofacial Surgery, College of Dentistry, King Khalid University, Abha, SAU

**Keywords:** atropine, asystole, bradycardia, surgery, maxillofacial trauma, occulocardiac reflex (ocr)

## Abstract

Oculocardiac reflex (OCR), presenting as bradycardia and asystole, is a potential intraoperative complication that may occur during maxillofacial trauma surgery. Bradycardia is the most common symptom of this phenomenon. Surgeons should be aware of its long-term effects, such as arrhythmias and even cardiac arrest. We report the case of a 40-year-old male patient with a fracture of the floor of the orbit. During a surgical exploration of the orbital floor, the patient exhibited sudden symptoms of OCR. It was managed by withholding the surgery and administering atropine. The article also highlights the mechanism, types, incidence, and management of OCR in patients with maxillofacial trauma.

## Introduction

The oculocardiac reflex (OCR) was first described by Dagnini and Ascher in 1908 as a pressure-induced neural reflex causing cardiac depression through vagal stimulation during strabismus surgeries [1,2]. The term "trigeminocardiac reflex" was then used by Shelly and Church in 1988 to refer to the overall mechanism. This word made it clear that while the OCR is most frequently triggered, it is only one specific manifestation of a more general reflex phenomenon [3]. Hence, there is a chance that any surgery performed on the trigeminal nerve's distribution could trigger a cardiovascular event in reaction to a trigeminal stimulus. The trigeminal and vagus nerves form the reflex arc known as the OCR. It is described as a heart rate reduction of more than 20% from baseline that occurs after extraocular muscle traction or globe manipulation [4]. The OCR has been observed during mandibular osteotomies and temporomandibular joint surgery. This highlights the possibility that the trigeminal nerve's maxillary and mandibular divisions could be involved as the ophthalmic branch [5]. In maxillofacial trauma, this occurrence is most common during the manipulation of the orbit and orbital contents during orbital floor fractures. The most common presentation is the herniation of the soft tissue contents of the orbit into the maxillary sinus. There can be entrapment of the ocular muscles causing restriction of ocular movements. In severe cases, ocular muscle entrapment, pain, edema, and compartment syndrome trigger OCR [6].

In this report, the authors describe a case of orbital floor fracture repair that triggered OCR in an adult male patient. The article also aims to discuss the pathophysiology, types, incidence, and management of OCR in maxillofacial trauma patients.

## Case presentation

**Figure 1:**
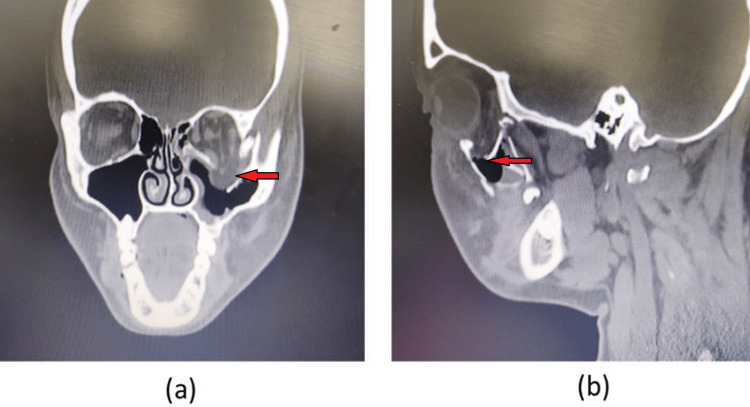
Preoperative CT images showing fracture of orbital floor (a) Coronal view; (b) sagittal view

A 40-year-old male patient presented with a history of interpersonal violence where he got punched in his left eye. The patient was conscious, oriented to time, place, and person, and had a Glasgow Coma Scale (GCS) of 15. Periorbital edema, subconjunctival hematoma, periorbital ecchymosis, and enophthalmos were the only positive findings. Pupil reflexes and ocular motility were intact. A CT scan was advised, which on examination revealed a fracture of the left orbital floor with the orbital soft tissue contents herniating into the maxillary sinus (Figure 1).

**Figure 2:**
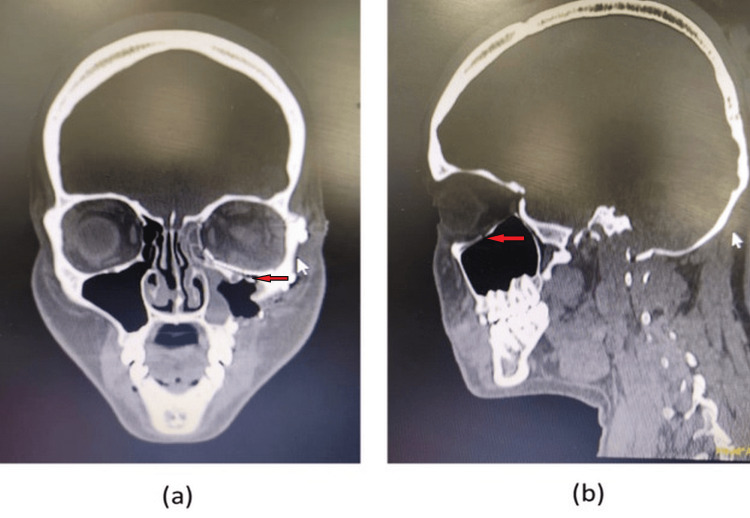
Postoperative CT images showing reduced and fixed orbital floor (a) Coronal view; (b) sagittal view

Open reduction and internal fixation under general anesthesia were planned. A forced duction test was performed, which showed no entrapment of ocular muscle into the fracture fragments. The surgical site was explored with an infraorbital incision; layered dissection was conducted to reach the rim and the floor. Bony fragments were manipulated, and the soft tissue contents were retracted to fill the orbital space. Reconstruction of the orbital floor with a titanium orbital mesh plate was planned. While manipulating the left globe, a sudden drop in heart rate of up to 30 beats per minute was observed. The surgery was stopped for a few minutes, during which the heart rate rose to 50 beats per minute. On further manipulation of the globe in an attempt to reconstruct the orbital floor, the heart rate again dropped to 22 beats per minute. Immediately, the surgery was withheld, and atropine was injected intravenously, 0.25 mg twice with a gap of two minutes by the anesthetist. A heart rate of 72 beats per minute was achieved. The reconstruction of the floor, maintaining the orbital soft tissue contents, was conducted as soon as possible. The patient remained hemodynamically stable and was soon weaned off by the effect of atropine after extubation. The patient was stable, conscious, and oriented postoperatively. A cardiology review was advised after three days, and no cardiac disease was identified. Figure 2 shows the postoperative CT scan images with the reconstructed orbital volume.

The patient had less postoperative discomfort on the fourth day and was completely stable to be discharged. The patient was kept on follow-up for up to 30 days, and no ill events were noted.

## Discussion

The rapid onset of parasympathetic dysrhythmia, sympathetic hypotension, apnea, or stomach hypermotility in response to the activation of any of the trigeminal nerve's sensory branches is known as the trigeminal-cranial reflex (TCR). It is thought that the sensory nerve endings of the trigeminal nerve form the afferent pathway of the reflex arc by sending neuronal signals to the trigeminal nerve's sensory nucleus through the Gasserian ganglion. This process is the mechanism behind the creation of the TCR. This afferent pathway connects to the efferent pathway in the motor nucleus of the vagus nerve by continuing along the short internuncial nerve fibers in the reticular formation [7]. The activation of stretch receptors in the periorbital and ocular soft tissues, either by direct traction or increased pressure, initiates the reflex, which is a subvariant of the TCR. Therefore, the vagal motor response is stimulated, sending impulses to the sinoatrial node and slowing the heart rate [8]. In reality, TCR is the brain's natural physiological defense system against ischemia. It is a reflex that conserves oxygen. Shortly after the onset of this reaction, sympathetic nervous system activation occurs, resulting in cerebral vascular vasodilation. These are exaggerated reactions that endanger the patient. The cardiac depression peaks during the first phase of vagal stimulation, which might result in ventricular fibrillation, asystole, or sinus arrest [9].

Trigeminocardiac reflexes come in two varieties: central and peripheral. Bradycardia, apnea, and hypotension are the symptoms of the central type, which is brought on by the activation of the Gasserian ganglion. The OCR and maxilla-mandibular cardiac reflex are two of the three subtypes of the peripheral type, which cause bradycardia, apnea, and normotension. The anterior ethmoidal nerve stimulates the third type, which is a diving response that causes bradycardia, apnea, and hypertension [10].

Lübbers et al. categorized facial surgeries into low risk (temporomandibular joint surgeries, LeFort I osteotomy, elevation of zygoma), medium risk (skull base surgeries), and high risk (ophthalmic surgeries, orbital exenteration, and orbit fracture in children with cardiac disease) based on the rate of incidence of TCR [5]. Any oral surgical procedure has the potential to cause TCR, so its significance should not be underestimated.

TCR/OCR secondary to trauma has been reported in midface disimpactions, elevation of the zygomatic arch, zygomaticomaxillary fracture fixations, naso-orbital-ethmoidal fracture fixations, and orbital floor reconstruction. In a retrospective study conducted by Shanab, 27.3% of midface trauma and 17.9% of lower-face trauma presented with TCR [11]. In one case out of two, reported by Oxley et al., a patient had a zygomatic arch fracture that was approached intraorally and showed bradycardia [12]. The authors stated that intraoral reduction of the arch can cause irritation and pressure to the temporalis muscle, which is directly involved in the reflex arc that caused the reaction. Moreover, as it was a case of delayed treatment, the authors believe that the partial union at the zygomatic bone might have occurred, which, on manipulation, could cause changes in the orbital pressure leading to the symptoms. In the second case, there was displacement of bony structures into the orbit and post-traumatic swelling, leading to compartment syndrome, which triggered the reaction. Brasileiro et al. reported a case of an adult female with a trapdoor type of orbital floor fracture [13]. The patient had no severe signs and symptoms of trauma. Mild subconjunctival hemorrhage, mild pain, and diplopia because of restriction of eye motility were the only findings. The patient complained of pain of eye motility leading to nausea, dizziness, and vomiting. Scans revealed mild disruption at the floor of the orbit with ocular muscle entrapment. On surgical manipulation, short-term bradycardia was seen, which was relieved after the muscular trap release. The authors advised that the presence of painful restriction in ocular motility associated with nausea or vomiting should alert the clinician to the possibility of an orbital “trapdoor” fracture with a significant risk for the development of hemodynamic changes [13]. In the present case, there were no signs of painful eye movements, restriction in motility, diplopia, and compartment syndrome. Excessive pressure and stretching while manipulating the globe and ocular musculature could be the reason for the sudden drop in heartbeats. The return of heartbeats to normal after discontinuation of the surgical manipulation was confirmatory. A similar experience is reported by Bhattacharjee et al. in their case study [10].

Hypercarbia, hypoxemia, insufficient anesthesia, and the nature of the stimulus are predisposing factors. It is more common in children because of high resting vagal tone. Light anesthesia, opioids, beta-adrenergic blockers, and neuromuscular blockers are also considered predisposing factors [14]. Most cases only result in a 10% to 50% drop in the heart rate, and when stimulation is stopped, sinus rhythm typically returns to baseline [15,16].

Reddy et al. [17] in their case report found a similar cardiac reflex in a child patient, which was associated with orbital floor fracture, suggestive of the importance of OCR. The OCR, characterized by bradycardia and other symptoms, is a potential complication in patients with maxillofacial trauma, particularly those with orbital floor fractures by Yoo et al. [18], Pham et al. [8], DesPain et al. [19]. Prompt recognition and management, including surgical intervention when necessary, are crucial to prevent morbidity and mortality [8,19]. The reflex can evolve over the patient's clinical course, underscoring the need for ongoing monitoring and intervention [18].

During maxillofacial trauma surgery, intraoperative cardiac investigations are essential in confirming the diagnosis of OCR. Real-time assessment of cardiac rhythm is possible through continuous electrocardiogram (ECG) monitoring, which can quickly identify bradycardia or asystole [20]. Additional information about cardiovascular health can be gained from pulse oximetry, which tracks oxygen saturation levels and looks for abrupt drops that could indicate problems with heart function. Similar to the implantation of an arterial line, invasive blood pressure monitoring provides continuous blood pressure monitoring, which is essential for detecting hemodynamic changes linked to OCR. With real-time assessment of structural abnormalities and cardiac function, transesophageal echocardiography (TEE) provides visualization of the heart's chambers, valves, and function. End-tidal CO2 levels are measured by capnography, which is significant during OCR-induced bradycardia or asystole as it indicates decreased cardiac output or perfusion. To treat electrolyte imbalances that put patients at risk for arrhythmias, it is essential to monitor serum electrolyte levels, especially potassium. The early detection and treatment of changes related to OCR are facilitated by the real-time data on hemodynamic parameters provided by continuous cardiac output monitoring techniques [21].

Developing a treatment algorithm for handling incidents of OCR during maxillofacial trauma surgery is essential. Upon recognition of OCR symptoms such as bradycardia or asystole, immediate intervention is crucial, involving temporary cessation of surgery, airway management, and atropine administration to block vagal stimulation. Further evaluation should include assessing electrolyte levels and optimizing fluid status, with consultation from specialists as needed. Once symptoms are managed, surgery can cautiously resume, with continuous monitoring and postoperative observation for any recurrence or complications, ensuring optimal patient care and outcomes [9].

## Conclusions

We strongly suggest considering TCR/OCR as the possible reason for bradycardia, hypotension, and asystole in cases of maxillofacial trauma, especially in the midface and the orbits. Immediate removal of the stimulus by stopping the surgery is advisable, along with judicious use of vagolytic agents. Prompt evaluation, monitoring, and management can save the patient from serious complications.
